# Assessment of a Culturally-Tailored Sexual Health Education Program for African American Youth

**DOI:** 10.3390/ijerph14010014

**Published:** 2016-12-24

**Authors:** Tiffany Zellner Lawrence, Tabia Henry Akintobi, Assia Miller, Elaine Archie-Booker, Tarita Johnson, Donoria Evans

**Affiliations:** 1 Department of Community Health and Preventive Medicine, Morehouse School of Medicine Prevention Research Center, 720 Westview Drive SW, Atlanta, GA 30310, USA; takintobi@msm.edu; 2 McKing Consulting Corporation, 2900 Chamblee Tucker Road, Building 10, Suite 100, Atlanta, GA 30341, USA; amiller@msm.edu; 3 Department of Community Health and Preventive Medicine, Morehouse School of Medicine, 720 Westview Drive SW, Atlanta, GA 30310, USA; eabooker@msm.edu; 4 Wholistic Stress Control Institute, Incorporated, 2545 Benjamin E. Mays Drive, Atlanta, GA 30311, USA; tjohnson@wholistic1.com; 5 ICF International, 3 Corporate Square NE Suite 370, Atlanta, GA 30329, USA; devans@icf.com

**Keywords:** African American, adolescents, sexual health, evaluation

## Abstract

African American youth are affected disproportionately by sexually transmitted infections (STIs), human immunodeficiency virus (HIV)/acquired immune deficiency syndrome (AIDS), and teenage pregnancy when compared to other racial groups. This paper evaluates the effectiveness of the To Help Young People Establish (2 HYPE) Abstinence Club, a behavioral intervention designed to promote delayed sexual activity among African American youth ages 12–18 in Atlanta, Georgia. The intervention included 20 h of curriculum and creative arts instruction. Pre- and post-intervention survey data collected from 2008–2010 were analyzed to determine the effectiveness of the intervention. Intervention (*n* = 651) and comparison (*n* = 112) groups were compared through analysis of variance and multivariate logistic regression models. There was a statistically significant increase in intervention youth who were thinking about being abstinent (*p* = 0.0005). Those who had not been engaged in sexual activity were two times more likely to plan abstinence compared to participants that had been previously sexually active previously (odds ratio 2.41; 95% confidence interval 1.62, 3.60). Significant results hold implications for subsequent community-based participatory research and practice that broadens the understanding of the relevance of marriage, as just one among other life success milestones that may hold more importance to African American youth in positioning the value of delayed and responsible sexual activity towards effective STIs, HIV/AIDS, and teen pregnancy risk reduction interventions.

## 1. Introduction

African American youth are affected disproportionately by sexually transmitted infections (STIs), human immunodeficiency virus (HIV)/acquired immune deficiency syndrome (AIDS), and teenage pregnancy when compared to other racial groups [1,2,3,4,5,6]. Despite teen pregnancy rates being at an all-time low in the United States, disproportionate rates still remain between African American, Hispanic, and White youth [1]. In 2014, the teen birth rate remained nearly twice as high for Hispanic and non-Hispanic Black teens compared with non-Hispanic White teens [1]. When compared to other races, African American youth account for a higher proportion of new HIV diagnoses, those living with HIV and those ever diagnosed with AIDS [2]. In fact, African American youth account for almost 60% of new HIV cases among persons aged 13–24 [2]. Similarly, chlamydia, gonorrhea, and syphilis rates are significantly higher in African American youth compared to Whites [3]. The disparities seen nationally are similar to rates seen in the state of Georgia [1,2,5,6,7].

In order to combat risky sexual behaviors in youth and mitigate negative outcomes, sexual health interventions were created. According to the Centers for Disease Control and Prevention (CDC), the best HIV risk reduction interventions will be rigorously evaluated and have significant and positive evidence of efficacy in areas such as eliminated or reduced sex- or drug-risk behaviors, reduction in the rate of new HIV/STIs and increased HIV-protective behaviors [8]. While these prevention approaches are employed in the United States and internationally, success greatly depends on cultural settings and community involvement [9]. While the approach by Green et al. [9] to increase abstinence showed great effectiveness internationally, the evidence about effectiveness of abstinence promotion interventions suitable for high risk African American youth based on community-based participatory research (CBPR) in the United States is limited.

A recent study found that youth that received sex education, regardless of the type, were associated with healthier sexual behavior, such as delayed sexual initiation and greater use of contraception [10]. A study which examined the impacts of four abstinence education programs found that there was no significant impact on teen sexual activity or rate of unprotected sex [11]. A systematic review conducted on abstinence-only education interventions indicated that there was a lack of rigor to demonstrate their effectiveness [12]. Lack of rigor in research methods is often cited as a limitation of abstinence education programs. Many of these programs are not designed as randomized clinical trials, and longitudinal follow-up is rarely implemented [11,12,13]. Behavioral intentions and change in attitudes take time to be transformed into behavioral changes, therefore, the long term effect could be underestimated [14]. For instance, studies which did utilize a randomized, controlled design [15,16] and longitudinal follow-up [15] demonstrated effectiveness in areas of intentions to maintain abstinence, delayed sexual initiation, and proabstinence attitudes. It is clear that additional research must be done in order to contribute to the literature on abstinence education effectiveness.

Given the alarming rates of disparities, we are in need of effective programs which not only target African American youth, but are tailored for them to increase their retention in programs and expectant changes in their behavior. The objective of this study is to assess the effectiveness of the To Help Young People Establish (2 HYPE) Abstinence Club in positively impacting attitudes in African American youth ages 12–18 regarding abstinence, marriage, and associated behavioral intentions to delay sexual activity.

### The 2 HYPE Abstinence Club

In an effort to reduce the rates of teen pregnancy, STIs, and HIV/AIDS among African American adolescents in Metropolitan Atlanta, the Wholistic Stress Control Institute, Inc., Atlanta, GA, USA (WSCI), designed and implemented the 2 HYPE Abstinence Club. WSCI is an African American non-profit community-based organization which has worked in the African American community for over 30 years providing community prevention programs related to HIV/AIDS, teen pregnancy, substance abuse, stress management, mental health, and violence. The 2 HYPE Abstinence Club, is a culturally-tailored abstinence education program for African American youth conducted between 2008 and 2010. The 2 HYPE Abstinence Club was designed to promote sexual abstinence until marriage. The program was designed to increase knowledge of STIs, sexual activity refusal skills and techniques, stress management, youth empowerment, self-control, self-esteem, and goal-setting. The 2 HYPE Abstinence Club is based upon three learning theories: Experiential Learning Theory [17], Social Learning Theory [18], and Cognitive Learning Theory [19]. The theories emphasize learning through personal experience or the experiences of others, learning from your environment, and modeling desired behaviors. The theories were incorporated into the 2 HYPE Abstinence Club through role play, creative arts activities, popular culture references, and group discussions. The Choosing the Best Curriculum [20] provided the primary abstinence educational content for the 2 HYPE Abstinence Club. The 2 HYPE Abstinence Club was culturally adapted through input from a youth advisory board, consisting of youth similar, in regards to age, race, and socioeconomic status, to those that would participate in the program. During a planning session, youth suggested that the program should include creative arts and recreational activities. In response to this, culturally-specific art forms, such as hip hop, West African dance, spoken word, and poetry, were incorporated into sessions to creatively reinforce prevention information and skills taught during the session. The intervention was delivered by trained WSCI health educators, who completed a three-day training on the Choosing the Best curriculum and were certified in abstinence education. The intervention consisted of 20 h of instruction including: (1) 16 h of abstinence education with an infusion of hip-hop/spoken word, poetry, art, and cultural activities simultaneously delivered to reinforce the abstinence messages; and (2) 4 h of life skills instruction in the areas of violence prevention, substance abuse prevention, stress management, and nutrition. Upon completion of the program, a booster session was held monthly, for a total of three months of post-programming. Table 1 details the sessions, descriptions, and objectives associated with the 2 HYPE Abstinence Club. In order to evaluate the 2 HYPE Abstinence Club, WSCI initiated a community-academic partnership with the Morehouse School of Medicine Prevention Research Center, Atlanta, GA, United States (MSM PRC) to serve as outside evaluators to the program. MSM PRC evaluations have been designed to address needs mutually identified by the funder, grantees, and target populations to assure that program activities (1) are audience-driven; (2) foster sustained ownership of data collection processes; (3) implement participatory processes; and (4) are perceived as central to program planning, implementation, and sustainability. Additional details on program formation and pilot results can be found elsewhere [21]. This study received an exempt review of evaluation methodology and instruments through the Morehouse School of Medicine Institutional Review Board in February 2008 (project identification code 281244-1).

## 2. Materials and Methods

### 2.1. Study Population

A total of 1026 youth between the ages of 12 and 18 were recruited between March 2008 and September 2010. WSCI health educators utilized a purposive sampling plan to recruit youth from seven schools, three community sites, and three detention sites in Metropolitan Atlanta, Georgia. Health educators held orientation at each site to discuss the curriculum and distribute informational packets and referral forms prior to program implementation. Each site participated in the 2 HYPE Abstinence Club as an intervention site, where youth would receive the full 2 HYPE Abstinence Club intervention, or a comparison site where youth would only complete pre- and post-test surveys and receive generalized health information for staff to distribute. Therefore, randomization by sites was used to assign youth to groups. Parental consent and youth assent were obtained from both intervention and comparison group participants.

Only youth from the population who completed both pre- and post-test surveys were used in the sample. Eight hundred and eighteen (818) youth completed both pre- and post-test surveys. This represents an 80% retention rate. To be eligible for the post-test, participants were required to have attended all core classes. The core classes included Choosing the Best Journey Lessons 1–8 and Choosing the Best Soul Mate Lessons 1–5 representing the key components of the intervention. Sociodemographic characteristics of participants that completed the post-test did not differ from those who were not eligible to take the post-test. Additionally, only youth that reported their racial identity as African American were included in this sample. A total of 763 youth matched these requirements. Among these youth, 651 participated in the 2 HYPE Abstinence Club while 112 youth were a part of the comparison group.

### 2.2. Measures

The MSM PRC administered confidential pre-test and post-test surveys to youth in intervention and comparison groups, approximately six to eight weeks apart. Only the intervention youth who completed all core curriculum sessions were eligible to take the post-test. Evaluation objectives were assessed through a questionnaire developed based on adaptation of two survey instruments [22,23]. Our final questionnaire presented good reliability and internal consistency (Cronbach’s α = 0.7508). Participant characteristics were measured through demographic items included on the survey. The primary outcome measure of the study was behavioral intention to abstain from sexual intercourse. Secondary outcome measures were attitudes and beliefs about sex and marriage.

#### 2.2.1. Behavioral Intention to Abstain from Sexual Intercourse Scale

Respondents were asked to respond to three statements regarding their behavioral intention to abstain from sexual intercourse: (1) ‘I plan to save sexual activity for marriage’ and (2) ‘I am thinking about being abstinent’. Responses included a five-point Likert scale ranging from ‘Strongly disagree’ to ‘Strongly agree’; Additionally, the statement (3) ‘Do you think you will have sex at any time before you get married?’ comprised responses “I'm certain I won’t”, “I probably won’t”, “I’m not sure whether I will or not”, “I probably will”, and “I’m certain I will”.

#### 2.2.2. Attitudes and Beliefs about Sex and Marriage Scale

A series of 10 statements were asked about participants’ attitudes and beliefs about sex and marriage. Statements included were (1) ‘It is important for me to wait until marriage to have sex’; (2) ‘Sexual urges can be controlled’; (3) ‘Even if I am physically mature, that doesn’t mean I’m ready to have sex’; (4) ‘I think it is okay to say “NO” when someone wants to touch me’; and (5) ‘Even if there is not pregnancy, having sex can cause a lot of problems for unmarried teenagers‘. Response options included “Agree”, “Disagree”, and “Not sure”; Additional statements included (6) “Would having sex as a teenager make it harder for someone to study and stay in school in the future?” and (7) “Would having sex as a teenager make it harder for someone to have a good marriage and family life in the future?” with response options ranging from “No, not harder at all” to “Yes, much harder” and “Haven’t thought about it yet”; Additionally, responses to statement (8) ‘Is there a problem with unmarried teens having sexual intercourse if no pregnancy results from it?’ comprised “No problem at all”, “Some problem”, and “A big problem”; to statement (9) ‘Do you feel comfortable talking to your girlfriend or boyfriend about the decision to not have sex?’ comprised “Yes”, “No”, “May be”; to statement (10) ‘How sure are you that you could keep from having sex?’ were “Not sure”, “A little sure”, and “Very sure”.

### 2.3. Analysis

Matched pre- and post-surveys were utilized for analysis. Survey responses were analyzed, comparing frequencies and means for each outcome variable from pre- to post-test and between intervention and comparison groups. We reported mean, standard deviation, and *p* values to determine the effect of the intervention on the outcomes of interest. A series of analysis of variance (ANOVA) were conducted. We used the generalized linear procedure in which each outcome measure was the dependent variable and time to collect data (pre-test = 0, post-test = 1), and study group (comparison = 0, intervention = 1) were predictors. The ANOVA model was controlled for potential covariates such as gender (male = 1, female = 2), grade (6th–8th grades = 1, 9th–10th grades = 2, 11th–12th grades = 3), and site of interview (school = 1, detention center = 2, community = 3).

We examined the behavioral intention to be abstinent (‘I am thinking about being abstinent’—“Disagree”, “Not Sure”, “Agree”) by predictors such as study group, time to collect data, school grade, site of the interview, gender, and engagement in sexual activity (‘Have you ever had sexual intercourse?’ “Yes” vs. “No”). Factors associated with the behavioral intention of being abstinent with *p* values < 0.05 in bivariate analyses were included in a multivariable model that used a multilevel outcome variable with the reply “Disagree” of being abstinent as the reference compared to reply for each of the other 2 levels (“Agree”, “Not Sure”). Statistical analyses were carried out using SAS version 9.2 (SAS Institute Inc., Cary, NC, USA).

## 3. Results

### 3.1. Sample Characteristics

A total of 763 youth completed pre-test and post-test surveys. As detailed in Table 2, there were 651 youth in the intervention group and 112 youth in the comparison group. One hundred percent (100%) of the youth sampled identified themselves as Black/African American. Ages ranged from 12 to 18 years, with a mean age of 15.0 years for the intervention group and 15.2 years for the comparison group. The mean grade of both groups was 9.3. The largest proportion (43.3%) of participants in the intervention group was between 9th to 10th grades. The largest proportion (35.2%) of participants in the comparison group were in 6th, 7th, or 8th grades. The largest proportion of youth in each group was female (54.9% intervention and 68.8% comparison). Over half (55.0%) of youth in the intervention group participated at school sites, whereas the majority (60.7%) of the comparison group was from community sites. Both groups reported a similar proportion of youth who had engaged in sexual activity prior to participating in the program (47.4% intervention and 46.6% comparison). While the intervention and comparison groups were statistically different with respect to grade-level (*p* = 0.034), gender (*p* = 0.007) and site of interview (*p* < 0.0001), they were not statistically different on program inclusion criteria characteristics (race and age).

### 3.2. Behavioral Intention to Abstain from Sexual Intercourse

Positive changes were observed for each of the questions associated with behavioral intentions to abstain from sexual intercourse (Table 3). We found that after the intervention there was a statistically significant increase in the number of youth who agreed that they were thinking about being abstinent (*p* = 0.0005). Additionally, post-intervention, more youth agreed that they planned to save sexual activity for marriage and were certain that they would not have sex any time before they got married.

We included factors that were significantly associated with the intention of being abstinent in univariate analysis in the multivariate multinomial logistic regression model (Table 4). Our final model revealed that at post-test, participants were more likely to plan to be abstinent compared to pre-test (odds ratio (OR) 1.41; 95% confidence interval (CI) 1.02, 1.95), males were two times more likely to plan abstinence compared to females (OR 2.00; 95% CI 1.45, 2.77), and participants who had not been engaged in sexual activity were two times more likely to plan abstinence compared to participants that had been sexually active previously (OR 2.41; 95% CI 1.62, 3.60).

### 3.3. Attitudes and Beliefs

Changes in attitudes and beliefs are presented in Table 5. After the intervention there was a significantly higher proportion of youth who agreed that sexual urges can be controlled (*p* = 0.0025). Additionally, a positive impact of the intervention was shown through a significant increase of agreement with “Is there a problem with unmarried teens having sexual intercourse if no pregnancy results from it?” (*p* = 0.0001) and “How sure are you that you could keep from having sex?” (*p* = 0.0464).

## 4. Discussion

The results of this study suggest that the 2 HYPE Abstinence Club was successful in participants gaining a better understanding of abstinence benefits. Intervention group participants demonstrated increases in all areas of the program including attitudes and beliefs, and, most important of all, in behavioral intentions related to abstinence. Though our evaluation showed statistically significant findings for certain outcomes, overall improvements that did not reach statistical significance are noteworthy and have a vital effect on local communities. Our findings are in agreement with a recent review presenting high-quality evidence that in-school interventions and interventions in geographically-defined communities designed for adolescents, can positively impact self-reported sexual risk behaviors and other HIV-related outcomes [24].

Findings of our study support the position that sexual health education programs seem to be more successful for youth that have not initiated sexual intercourse. These youth were shown to be more likely to intend to abstain. It is well known that behavior change can be a long, arduous process. According to the Stages of Change Model [25] youth that already initiated sex that wish to change their behavior would have to work through three stages to get to taking action and four stages to get to maintaining their new action, whereas youth that are already practicing a behavior (abstaining from sex) can be considered to be in the maintenance phase. This may be why abstinence education programs work more often for these youth. Abstinence education programs help those that have not initiated sex maintain their abstinence by reinforcing their attitudes and beliefs, and providing them with added tools and skills in which to maintain their status.

Though community-based participatory research approaches are being used broadly in research focused on prevention of a variety of health issues, its use in the development of sexual health interventions targeting African American youth is less understood. Community academic partnerships to both implement and evaluate promising interventions, can play a central role in developing central foci and determining contexts central to their effectiveness through position prioritizing communities, in this case, youth, as senior partners in intervention planning, implementation, and evaluation toward increased ownership and sustainability due to cultural and contextual relevance and responsiveness [26,27,28,29,30,31].

### 4.1. Acceptance of Abstinence-Until-Marriage

Even though the 2 HYPE Abstinence Club was shown to be successful in increasing knowledge about abstinence and changing youth’s attitude about abstinence there was not a broad acceptance of abstinence among youth participants. While there was a statistically significant increase in the number of youth thinking about being abstinent, less than half of participants were represented. Similarly, less than half of the youth thought that remaining abstinent until marriage was very important. One of the possible explanations for a low proportion of participants thinking about being abstinent could be the emphasis that abstinence and abstinence education programs place on marriage, rather than, more broadly, delayed sexual activity until one is more mature, responsible, and has achieved other life goals. It seems that youth may be thinking about abstinence or delayed sexual activity, but not necessarily about marriage.

### 4.2. Marriage as a Success Indicator

Future abstinence programs must work to address youth that may not believe they will get married in the future or simply have no desire to get married “right now”. Youth that come from divorced and single-parent homes have lower expectations to marry and have weaker support for marriage [32]. If an adolescent was raised in a single-parent household that was relatively stable, and they did not encounter perceived obstacles in this family structure, then they may not see the value in getting married. Hence, being raised in a two-parent/caregiver home where partners are married may not be perceived as a determinant of their life success.

Programs that solely correlate abstinence-until-marriage with delayed or protected sex in order to decrease STIs, HIV/AIDS, and teen pregnancy rates among African American youth should be considered in light of the social and cultural realities of the audience targeted. In this study, youth’s attitudes toward marriage were related with their intention to abstain. Youth that did not feel that marriage was an important goal reported that they would abstain from sex before marriage less than youth that felt marriage was important. Adolescents who participate in an abstinence-until-marriage intervention and do not intend to get married may, resultantly, be less likely to delay sexual activity if no alternatives towards delayed initiation are presented. For instance, youth delaying sex until after completing their education or becoming financially stable could be among intervention foci positioning youth toward future life success towards both delayed and responsible sexual behavior.

### 4.3. Strengths

This study has demonstrated strengths in several areas. First, we were able to recruit and retain a high-risk, vulnerable population of African American youth through our program. Second, this study’s use of a program which was culturally tailored and age appropriate for its specific population was a strength. More specifically, the educational curriculum was developed using a community-based participatory approach which engaged youth to advise the form and content of the intervention to ensure cultural and age-specific resonance. Using the 2 HYPE Abstinence Club which specifically tailored the abstinence curriculum and program components to the target population was a benefit. Culturally-tailored programs are shown to be more effective [33]. Third, this study’s use of an intervention and comparison group to assess effectiveness strengthened this investigation. By using a comparison group that was similar to the intervention group, it is highly likely that the results observed were attributable to the intervention. Previous research was limited because a comparison group was not used to test program effects against an intervention group [34]. Finally, there was low attrition of participants, with 80% retained from pre- to post-intervention. This is likely attributable to the CBPR-driven approach and health educators who were socially and culturally congruent. Furthermore, pre-test and post-test matching by non-personal identifiers yielded results that were likely stronger than if anonymous survey administration had been implemented.

### 4.4. Limitations

This study utilized a purposive sample of program participants, recruited at local sites in Metropolitan Atlanta; therefore, the results are not generalizable to individuals that did not meet the program inclusion criteria. Our study relied on self-reported data from teens about a sensitive subject. Therefore, responses are subject to social desirability bias. Teens may have responded in a way they thought would be favorable, rather than how they truly felt. This study’s use of randomization by site is also a potential limitation. Site-level factors could influence the results that were seen in the program. Randomization at the individual level would be a valuable step. Additionally, although in our analysis we controlled for the statistical difference between the intervention and comparison groups this difference is possibly limiting. In addition, using a longitudinal design which follows participant’s long-term will enhance findings, by providing data on an individual’s change over time. Behavior change is a process and may not be seen immediately following a program, especially when speaking of adolescent sexual behavior, which is usually not a regularly occurring behavior. Denny and Young [13], hypothesized that abstinence programs may not be indicating significant, positive results because they do not conduct program follow-up [13]. They performed an evaluation of an abstinence education program which did an 18-month follow-up. The researchers found that at post-test there were no differences between treatment and control groups relative to behavior, but at 18-month follow-up there were significant differences between the two groups. The treatment group was less likely to report sexual intercourse in the last month when compared to the comparison group. In a randomized control study of a theory-based abstinence intervention that did a 24-month follow up researchers found significant differences between treatment and control groups [15]. A significant reduction in the number of partners was seen in comprehensive treatment groups when compared to the health promotion control group. Using a rigorous design in the future can fully assess the effectiveness of abstinence education programs not only for African American youth, but for all youth who participate in these programs.

### 4.5. Future Research

Broadly recognized heterogeneity in community and family contexts are acknowledged and may serve as important foci for future studies. This investigation was based upon three learning theories emphasizing learning through personal experience, the experiences of others, learning from one’s environment, and modeling desired behaviors. As among the first culturally- and CBPR-tailored studies prioritizing African American younger youth, discussing the thought processes of others and their lived experiences, overall, was an important benefit for youth in both groups, whether they have initiated sexual activity or not. Subsequent investigations focusing on important subgroups of younger African American youth, ages 10–14 are important [35] and may require different approaches and reap different results. Unique interventions targeting those who had not or who had initiated sexual activity, respectively, are important and potentially unique research paths. Additionally, given the larger proportion of youth (80%) acquiring sexually transmitted infections, such as HIV through male-to-male sex [2], further investigation on abstinence toward future life milestones that does not include marriage is critical.

## 5. Conclusions

The results of this study are promising and present implications for future research and practice that broadens the understanding of the processes and outcomes associated with effective interventions designed to reduce HIV, STIs, and pregnancy among African American youth. First, findings expand previous research through practical application and infusion of not only racial and ethnic, but life course realities and priorities of youth, as key intervention components, resulting in significant effects. Second, this study signals the strength of subsequent longitudinal, mixed-method community based participatory investigations that assess the relevance of marriage, as just one among other culturally-relevant life success milestones to set towards positioning the value of delayed sexual activity in risk reduction programs. In this study, youth attitudes toward marriage were related with their intention to abstain; and the intention to abstain was also strongest among youth who had never had sex. This study thereby contributes to the evidence critical to reducing sexual risk and outcome disparities among African American youth.

## Figures and Tables

**Table 1:** 2 HYPE Abstinence Club Curriculum Outline.

Session	Description	Learning Objectives
**Session 1**	Program Introduction	
**Session 2**	Hip-Hop; Spoken Word; West African Dance	
**Session 3**	Poetry; Art; Nutrition	
**Session 4**	Pre-Test Survey Administration	
**Session 5**	Choosing The Best Journey Lesson 1: Setting Goals Lesson 2: Making Best Decisions	What is a goal Avoiding Detours What is Determination Alcohol, Sex and Date Rape How to Make Decisions What is Wisdom
**Session 6**	Choosing The Best Journey Lesson 3: Avoiding Pregnancy Lesson 4: Avoiding Sexually Transmitted Diseases (STDs)	Reducing Risks Budgeting for Babies Responsibilities of Parents Common STDs Safe Sex What is Honesty
**Session 7**	Choosing The Best Journey Lesson 7: Overcoming Pressure	Overcoming Pressure Media Pressures What is Self-Discipline
**Session 8**	Choosing The Best Soul Mate Lesson 2: Being the Right One	What Guys and Girls are looking for in a Mate Increasing Self Confidence Understanding Temperaments Building on Strengths
**Session 9**	Choosing The Best Soul Mate Lesson 1: Finding the Right One	Avoiding the Five Relational Traps Avoiding the Sex Trap Six Keys to Finding and Keeping Your Soul Mate
**Session 10**	Choosing The Best Soul Mate Lesson 4: Dating to Discover	Dating to Discover Ten Key Compatibility Areas Character Traits
**Session 11**	Choosing The Best Journey Lesson 8: Being Assertive	Developing Refusal Skills Developing Assertiveness Skills What is Courage
**Session 12**	Choosing The Best Journey Lesson 5: Developing Best Relationships Choosing The Best Soul Mate Lesson 3: Developing Rational Skills	Avoiding Relational Traps Building Self Esteem What is Self-Respect Opposites Attract The Need for Versatility Developing Relational Skills Improving your Versatility
**Session 13**	Choosing The Best Journey Lesson 6: Choosing Abstinence Until Marriage Choosing The Best Soul Mate Lesson 5: Making Marriage Work	Choosing Abstinence Until Marriage Emotional Consequences Compatibility, Character, and Commitment Making Healthy Choices Making Marriage Work Packing Your Marriage Survival Kit Communication Challenges Improving Listening Skills Improving Appreciation Skills The Fact About Living Together Ten Commitments that Keep a Marriage Strong
**Session 14**	Violence Prevention; Substance Abuse Prevention; Stress Management	
**Session 15**	Guest Speaker; Hip-Hop; Spoken Word; Poetry; West African Dance	
**Session 16**	Post-Test Survey Administration; Graduation	

**Table 2:** Demographic characteristics of intervention and comparison groups at pre-test.

Characteristic	Intervention		Comparison		*p*-Value
	*n* (651)	%	*n* (112)	%	
**Age (years)**					
Mean ± SD	15.0 ± 1.7		15.2 ± 1.9		0.259
≤14	268	41.2%	41	36.6%	0.173
15–16	233	35.8%	36	32.1%	
≥17	150	23.0%	35	31.3%	
**Grade (6th–12th)**					
Mean ± SD	9.3 ± 1.8		9.3 ± 1.8		1.000
6th–8th	168	26.2%	38	35.2%	0.034 *
9th–10th	278	43.3%	33	30.6%	
11th–12th	196	30.5%	37	34.3%	
**Gender**					
Male	293	45.1%	35	31.3%	0.007 *
Female	357	54.9%	77	68.8%	
**Hispanic/Latino**	9	1.4%	1	0.9%	1.000
**Site of Interview**					
School	358	55.0%	34	30.4%	<0.001 *
Community	249	38.2%	68	60.7%	
Detention Center	44	6.8%	10	8.9%	
**Ever had sexual intercourse (yes)**	304	47.4%	52	46.6%	0.960

* Statistical significance was set at *p* ≤ 0.05; SD: standard deviation.

**Table 3:** Generalized linear model for behavioral intention to abstain from sexual intercourse.

Question	Group	Pre-Test Mean ± SD	Post-Test Mean ± SD	Adjusted † *p* Value
I plan to save sexual activity for marriage.	Intervention	2.94 (1.27)	3.02 (1.25)	0.1388
	Comparison	3.26 (1.33)	3.36 (1.22)	
I am thinking about being abstinent.	Intervention	3.19 (1.20)	3.59 (1.09)	0.0005 *
	Comparison	3.69 (0.97)	3.82 (0.95)	
Do you think you will have sex at any time before you get married?	Intervention	3.45 (1.34)	3.37 (1.37)	0.6695
	Comparison	3.09 (1.13)	3.30 (1.16)	

† Adjusted for gender, grade, and site of interview; * Significant at *p* value < 0.05.

**Table 4:** Multivariable multinomial logistic regression to predict behavioral intention to abstain.

Predictor	Outcome *	OR	95% CI
Study Group: Intervention vs. Comparison	Agree vs. Disagree	0.66	(0.39, 1.13)
	Not Sure vs. Disagree	0.72	(0.43, 1.21)
Time: Post-test vs. Pre-test	Agree vs. Disagree	1.41	(1.02, 1.95)
	Not Sure vs. Disagree	1.33	(0.97, 1.81)
School Grade: 6th–8th vs. 11th–12th	Agree vs. Disagree	0.86	(0.44, 1.69)
	Not Sure vs. Disagree	1.08	(0.56, 2.07)
School Grade: 9th–10th vs. 11th–12th	Agree vs. Disagree	0.83	(0.49, 1.40)
	Not Sure vs. Disagree	0.87	(0.53, 1.42)
Site of Interview: School vs. Community	Agree vs. Disagree	0.64	(0.35, 1.16)
	Not Sure vs. Disagree	0.96	(0.52, 1.75)
Site of Interview: Detention Center vs. Community	Agree vs. Disagree	1.46	(0.55, 3.87)
	Not Sure vs. Disagree	0.83	(0.29, 2.33)
Gender: Male vs. Female	Agree vs. Disagree	2.00	(1.45, 2.77)
	Not Sure vs. Disagree	1.32	(0.97, 1.80)
Sexual Activity: Never had sexual intercourse vs. Had sexual intercourse	Agree vs. Disagree	2.41	(1.62, 3.60)
	Not Sure vs. Disagree	1.26	(0.85, 1.85)

* Reference = Disagree; CI: confidence interval; OR: odds ratio.

**Table 5:** Generalized linear model for attitudes and beliefs about sex and marriage.

Question	Group	Pre-Test Mean ± SD	Post-Test Mean ± SD	Adjusted † *p* Value
It is important for me to wait until marriage to have sex	Intervention	2.06 (0.89)	2.04 (0.91)	0.6427
	Comparison	1.85 (0.94)	1.73 (0.88)	
Sexual urges can be controlled	Intervention	1.68 (0.87)	1.54 (0.82)	0.0025 *
	Comparison	1.57 (0.82)	1.47 (0.80)	
Even if I am physically mature, that doesn’t mean I’m ready to have sex	Intervention	1.50 (0.77)	1.49 (0.77)	0.6491
	Comparison	1.55 (0.78)	1.49 (0.78)	
Would having sex as a teenager make it harder for someone to study and stay in school in the future?	Intervention	2.22 (1.09)	2.30 (1.08)	0.2368
	Comparison	2.41 (1.20)	2.44 (1.17)	
Would having sex as a teenager make it harder for someone to have a good marriage and family life in the future?	Intervention	1.70 (0.71)	1.76 (0.66)	0.1344
	Comparison	1.78 (0.78)	1.77 (0.72)	
Is there a problem with unmarried teens having sexual intercourse if no pregnancy results from it?	Intervention	2.03 (0.66)	2.16 (0.63)	0.0001 *
	Comparison	2.17 (0.70)	2.18 (0.69)	
Do you feel comfortable talking to your girlfriend or boyfriend about the decision to not have sex?	Intervention	1.45 (0.75)	1.28 (0.65)	0.1417
	Comparison	1.40 (0.75)	1.60 (0.91)	
I think it is okay to say “NO” when someone wants to touch me	Intervention	1.31 (0.60)	1.27 (0.58)	0.1094
	Comparison	1.28 (0.57)	1.21 (0.54)	
How sure are you that you could keep from having sex?	Intervention	2.21 (0.83)	2.38 (0.78)	0.0464 *
	Comparison	2.36 (0.78)	2.53 (0.80)	
Even if there is not pregnancy, having sex can cause a lot of problems for unmarried teenagers	Intervention	1.32 (0.63)	1.32 (0.66)	0.8921
	Comparison	1.28 (0.62)	1.23 (0.60)	

† Adjusted for gender, grade, and site of interview; * Significant at *p* value < 0.05.
